# Group Infection by the Bacillus Suipestifer

**Published:** 1920-08-21

**Authors:** 


### A RARE CASE OF FOOD POISONING.

### Group Infection by the Bacillus Suipestifer.

In view of the somewhat inconsistent reports lately published concerning a case of food-poisoning at Brixton, when ten members of a household were affected, one succumbing to the effects of the poison, the following account obtained direct by an interview with the Medical Officer of Health is of considerable interest. Great credit is due to Dr. Joseph Priestley for his thoroughness in dealing with this outbreak and showing to a remarkable extent what can be done by a careful and painstaking investigation of all the facts.

### The Medical History.

The house was inhabited by a family of seven people, together with two lodgers and a lady visitor staying for the week-end, making ten persons, all of whom were affected by symptoms which were described by the doctor in attendance as feverishness, rapid pulse, sickness and violent colicky pains. No single member of the house escaped. Some were taken ill on the early morning of Sunday, July 25, others in the early evening of the same day, and others again late in the evening, whilst the father of the family was not taken ill until Monday, July 26, at midnight. His wife was ill on the previous Thursday, and had a relapse or second attack on Sunday. Consequently it became clear that the poison was being partaken of over a period of twenty-four hours.

The food examined included boiled haddock, which was served with parsley butter, a stew of steak and liver, and a joint of roast ribs of beef, together with rock cakes. The latter were made with dripping bought from a stall in the New Cut, Lambeth, on the Saturday. By a process of exclusion, Dr. Priestley came to the decision that the only food that appeared to be common to the whole household was the steak-and-liver stew, and that the case was complicated by the fact that the gravy from this, which had been prepared on the Saturday for the midday meal, was warmed up and used as gravy with the roast beef on Sunday, which was cooked and eaten at dinner on that day.

"A rough bacteriological examination was made," continued Dr. Priestley, "of the various articles of diet, which disclosed that the steak-and-liver gravy juice contained what is known in bacteriological literature as the *Bacillus coli*, which is a normal inhabitant of the alimentary canal of man and animals, showing that the particular steak-and-liver gravy juice had become in some way infected with fæcal contamination, either from a human being or from an animal. A closer examination of the juice has shown it also to contain a germ which has been associated on previous occasions with outbreaks of food-poisoning, more especially on the Continent. This germ is known as the *Bacillus suipestifer*, and is one of the Gärtner group."

Dr. Priestley said there is no doubt that the food was infected by this particular germ, with the result that the whole of those who partook of the food were affected, and one, unfortunately, had died. The same germ was found in all the organs of the man's body, which since the post-mortem examination were submitted to careful and detailed bacteriological examination at the Lister Institute.

The question then arose as to where the germ originally came from. Dr. Priestley ascertained that the mother of the family was taken ill on Thursday, July 22, with an acute attack of sickness and diarrhœa and colicky pains, having been to Margate on the previous Sunday with her husband by boat, returning the same day. She did not go to bed, but continued throughout the week to do her ordinary domestic work and cooking for the family.

### The Explanation.

"The theory that finds most favour with me," added Dr. Priestley, "is that in all probability this lady was a carrier, and that the germ in question was the particular one mentioned, and that it was inoculated by personal contact with the steak-and-liver stew during its preparation. Such a stew being a suitable medium or nidus for this germ, the latter grew and multiplied in large numbers, so that the steak-and-liver juice was the most virulent poison. One strange fact is noteworthy, namely, that the father of the family was at work on Sunday and did not partake of the roast beef, gravy, and Yorkshire pudding that was served up to the family on that day, but he was provided with a pudding made from the steak and liver, and this he took to work, where it was thoroughly cooked (sterilised), with the result that it had no bad effect on him. But when he returned home and partook of the beef and Yorkshire pudding, with its accompanying gravy, on Monday midday, he was taken with a violent attack late on Monday night. Even his wife, who had had an acute attack on the Thursday, ate a little of the beef and Yorkshire pudding and the gravy on the Sunday, with the result that she had a second attack late on Sunday night. The liver and meat were sound and fit for human consumption when purchased from the butcher. Other people who had eaten the same had not suffered, and the liver was sold to a coffee-house keeper, where thirty or forty persons must have had some, and yet there is no complaint of any illness."

"It is only fair to say," continued Dr. Priestley, "that the house where the outbreak occurred is clean and sanitary, and that the utensils used for cooking were also clean, and there is no possible blame attaching to the mother, who looked after the welfare and did the cooking for her family and guests."

"From time to time these cases," concluded Dr. Priestley, "in the form of an outbreak occur, and there are about 120 cases on record which have been investigated during the last twenty years. They occur mostly on the Continent, especially in Germany, and sometimes happen in England; and whilst the cause of the outbreaks have been suspected to be due to food infected by the bacillus I have mentioned, or a bacillus belonging to the Gärtner group, there is no other case that has been reported in which the facts have been able to be collected so exactly as in this case.

